# Comparative analysis of breast cancer characteristics in young premenopausal and postmenopausal women in Ghana

**DOI:** 10.1038/s41598-024-52129-w

**Published:** 2024-02-01

**Authors:** Kingsley Bosompem, Joseph Yorke, Tonnies Abeku Buckman, Samuel Gyasi Brenu, Michael Nyantakyi, Francis Somiah-Kwaw Aitpillah, Ishmael Kyei, Michael Ofoe Adinku, Dennis Afful Yorke, Christian Obirikorang, Emmanuel Acheampong

**Affiliations:** 1 Directorate of Surgery, Komfo Anokye Teaching Hospital, Kumasi, Ghana; 2 Department of Surgery, School of Medicine and Dentistry, Kwame Nkrumah University of Science and Technology, Kumasi, Ghana; 3 Department of Medical Laboratory Science, KAAF University College, Buduburam, Ghana; 4 Department of Molecular Medicine, School of Medicine and Dentistry, Kwame Nkrumah University of Science and Technology, Kumasi, Ghana; 5 Department of Medical Diagnostics, Kwame Nkrumah University of Science and Technology, Kumasi, Ghana; 6 Leicester Cancer Research Centre, Department of Genetic and Genome Biology, University of Leicester, Leicester, LE1 7RH UK

**Keywords:** Biochemistry, Cancer, Cell biology

## Abstract

Breast cancer is increasingly common among young women in Ghana. BCa is heterogeneous with unique traits that impact causes, prognostic, and predictive outcomes of patients before and after menopause. However, limited evidence exists on differences between young premenopausal (YPM) and postmenopausal cases in Ghana. This study compared breast tumour characteristics between YPM women (under 35 years) and postmenopausal women. We conducted a prospective cross-sectional study involving 140 BCa-diagnosed women at the Breast Care Clinic of Komfo Anokye Teaching Hospital (KATH), Kumasi from November 2019 to June 2021. Thirty-one (22.1%) of participants were YPM and 109 (77.9%) were postmenopausal. The median ages for YPM and postmenopausal were 32.0 (range: 25.0–35.0) and 57.0 (48.0–86.0) respectively. Invasive carcinoma was the most common histological type (97.1%). Left tumour location was the most frequent in both groups (51.6% for YPM and 51.8% for postmenopausal). Lumps detected were frequently in the outer upper quadrant in both groups (61.3% and 56.0%). The majority of the YPM women (80.7%) and postmenopausal women (87.0%) had stage III and IV diseases. Most YPM (64.5%) and postmenopausal women (64.4%) exhibited triple-negative breast cancer (TNBC). Both YPM 13 (56.6%) and postmenopausal participants 40 (56.3%) exhibited a predominantly partial response to neo-adjuvant chemotherapy but YPM women (21.7%) experienced disease progression than the postmenopausal women (12.7%). The study highlights consistent tumour characteristics and advanced clinical stages at diagnosis in both groups with a higher prevalence of TNBC. TNBC and HER2+ subtypes respond better to Anthracycline-based neoadjuvant chemotherapy. Establishing Breast Care Clinics in district and regional hospitals for early detection is crucial and further studies are warranted to understand the higher TNBC prevalence in black Africans and re-evaluate breast education programs to address the persistently late presentations.

## Introduction

Breast cancer (BCa) is the most prevalent global malignancy among women, with projections, indicating over 2 million new cases annually by 2030^1,2^. This global trend is mirrored in sub-Saharan Africa, particularly in countries like Ghana and Nigeria, where BCa is the most frequently diagnosed cancer in females^3,4^. However, this surge in incidence in Sub-Saharan Africa is paralleled by poor prognosis, leading to an exponential rise in BCa mortality rates^5,6^. In contrast, developed countries have seen a steady decline in death rates since the 1990s due to early detection programs and improved treatment options^7^. A striking feature is that the majority of African women seek treatment when the disease is advanced with over 70% of cases in Ghana, Kenya, and Nigeria being diagnosed at advanced stages, often involving aggressive subtypes^8–10^.

Globally, there is an increasing incidence of BCa in younger women. These younger women exhibit distinct tumour characteristics compared to older women, such as more aggressive tumours with larger sizes, higher disease grades, positive lymph nodes, and hormone receptor negativity^11,12^. They also tend to have higher proportions of triple-negative breast cancer (TNBC) molecular subtype followed by human epidermal growth factor receptor 2 (HER2) which is linked with unfavourable prognoses^12^. Anastasiadi et al. reported young women with non-metastatic breast cancer have a higher 5-year risk of developing metastatic breast cancer when compared to their older counterparts^13^. The disparities in tumour characteristics between young and older women may be attributed to variations in risk factors such as race or ethnicity, body size, and reproductive history^13,14^. Tumour characteristics and biological markers play a crucial role in predicting outcomes in different age groups^14,15^.

Breast cancers in women under 35 years are classified with a category of malignancies referred to as “younger pre-menopausal women's cancers”^16–19^. The available literature is limited due to the scarcity of comprehensive studies and variations in the definition of "young” which may encompass ages such as 30 years, < 35 years, < 40 years, or all pre-menopausal patients. Nevertheless, the majority of studies in global literature concur that a woman under the age of 35 is generally considered “young”^16–19^. Findings emerging from various studies in Ghana at Komfo Anokye Teaching Hospital (KATH)^20^ and Korle-Bu Teaching Hospital^21^ as well as other regions in Ghana^22^, demonstrate that majority of breast cancer cases occurred in premenopausal and perimenopausal women, concomitant with advanced disease and unfavourable prognostic features.

The Breast Care Clinic at KATH registers on average 200 new cases of BCa annually. In 2019, 216 cases of tissue diagnosed BCa were recorded Breast cancer registry at KATH. Approximately, 13% of these cases affected YPM women (35 years and below), while 50% impacted women aged 49 and older^23^. Currently, no published study has compared tumour characteristics between these age groups in Ghana. Additionally, there is a lack of epidemiological data on BCa tumour characteristics at KATH. The study seeks to examine and assess the trend in these characteristics in the above age groups and provide valuable epidemiological information for BCa management in KATH potentially influencing treatment guidelines and enhancing outcomes in these patients.

## Methodology

### Study design and setting

This hospital-based prospective cross-sectional study was conducted at the Breast Care Clinic (BCC) and the Oncology Department within Komfo Anokye Teaching Hospital (KATH) from November 2019 to June 2021. KATH, the second-largest hospital in Ghana, is located in the Ashanti region, Kumasi, with a thousand-bed capacity. KATH houses skilled medical professionals including doctors, nurses, anaesthetists, and specialized departments like surgery, internal medicine, obstetrics, gynaecology, paediatrics, oncology, family medicine, and emergency care. It is strategically located, providing excellent accessibility for patients from the Ashanti Region and beyond. The BCC at KATH is discreetly positioned opposite the Human resource directorate of KATH. The BCC at KATH was established in July 2004 to take care of all patients with breast complaints. The clinic offers an autonomous daily service exclusively focused on breast diseases. The clinic days are handled by five surgical specialists. The clinic has a staff strength of ten (10): five registered nurses, two enrolled nurses, a health care assistant, and a community health nurse, led by a deputy chief nursing officer. All patients presented at the BCC undergo thorough evaluation including detailed medical history and complete clinical breast examinations. All breast lumps are assessed by a combination of clinical examination, and breast imaging (breast ultrasound and or mammography).

### Study population and subject selection

The study enrolled all young pre-menopausal (YPM) women, defined as women aged 35 years and below and postmenopausal women with a histological confirmed BCa diagnosis presenting at Breast Care Clinic, KATH. The choice of the 35-year age threshold was made by harmonizing the global literature variations of different age cut-offs, such as < 30 years, < 35 years, < 40 years or all pre-menopausal used to define “young” patients with the majority considering under 35 as “young”^11,17–19^. Postmenopausal status was defined as a woman at least 48 years old with 12 consecutive months without menstruation. Patient selection was done using a convenience sampling approach and it took place in the consulting room every day of the week, starting from 8 a.m. to 2 p.m. during the study period.

### Data collection and tools

Data collection involved face-to-face interviews using a structured questionnaire with women diagnosed with BCa at the BCC. The questionnaire covered demographics, BCa risk factors, breast examination findings, histological features, and staging investigations. The principal investigator and trained assistant conducted the interviews. The questionnaire was written in English but was carefully translated into the local language to ensure clarity. It was developed with information from literature and guidance from supervising consultants and pretested with 12 BCa patients at the BCC. Problems identified from the pretesting phase were corrected and the final draft was prepared for collection of data. The interviewer administered the questionnaires during the study period.

### Inclusion and exclusion criteria

All women in the above age group presenting at the breast clinic with histologically proven diagnoses of breast adenocarcinoma received adriamycin and cyclophosphamide chemotherapeutic agents and consented to the study. Excluded from the study were patients with non-epithelial BCa, individuals with recurrent BCa, those prior incomplete breast mass removal, women unsuitable for chemotherapy (e.g., due to cardiac issues or early pregnancy with BCa), surgically induced menopause, cases of bilateral BCa, those undergone hysterectomy, experienced ovarian issues, lack specified menopausal status and those who decline consent.

### Study sample size

In 2019, among 216 cases of breast carcinoma diagnosed in women at BCC at KATH, 28 occurred in women under 35, and 108 occurred in those aged 49 years and above. The sample size for the study was determined using the Yamane formula which is suitable when the entire population (N) is known. In this instance, the required sample size was derived from a total of 216 cases resulting in a calculated sample size of 133. To account for a 5% attrition rate and increase statistical power, the sample size was adjusted to include 140 women.

### Laboratory estimation of immunohistochemistry

Specimens for histological diagnosis included core, excision, and incisional biopsies. All tissue specimens were kept in a 10% neutral buffered formalin solution within 48 h to 72 h. All biopsies were initially evaluated by routine hematoxylin and eosin-stained slides. Immunohistochemistry analysis was performed for all cases as part of routine assessment before initiation of chemotherapy. The measurement of tumour sizes before and after chemotherapy treatment was performed as part of the study. Histological grading was performed using the Scarff-Bloom-Richardson classification as modified by Elston-Ellis^24^. Tumors were staged according to the TNM system and regrouped using the American Joint Committee on Cancer Stages (AJCC 2010)^25^. Immunohistochemistry analysis for ER, PR, and HER2/neu expression was performed on the Ventana BenchMark GX autostainer. Both ER and PR were Rabbit monoclonal antibodies, ER clone SP1 and PR 1E2. The HER2 antibody used in the analysis was the Rabbit monoclonal antibody, 4B5. ER/PR positivity was defined as 1% nuclear staining following CAP/ASCO, 2020 recommendation. HER2/neu expression was categorized as per Fitzgibbons et al.’s recommendation^26^. Any specimen scored as 0 or 1+ was classified as HER2/neu negative, and specimens scored as 3+ were classified as positive. Cases with a score of 2+ were classified as equivocal, and reflex fluorescent in situ hybridization was used to assess the HER2/neu gene amplification. Fluorescent in situ hybridization for HER2/neu gene amplification was interpreted in compliance with ASCO/College of American Pathologists guidelines, and cases were reclassified as gene amplified or negative. Breast lump sizes were measured using a caliper and the longest diameter was recorded in centimeters. To assess tumour response to neoadjuvant chemotherapy, a follow-up measurement of any residual lump was taken a caliper (in centimeters), approximately two weeks after the completion of the fourth cycle of chemotherapy, following RECIST Criteria^27^.

### Ethical consideration

The study was approved by the Human Research, Publication, and Ethics (CHRPE/AP/68/19) of the School of Medicine and Dentistry (SMD), Kwame Nkrumah University of Science and Technology (KNUST) and the Research and Development (R&D) unit of KATH. Prospective participants, including those with no educational background, had their legally authorized representatives or literate guardians provide informed consent for their participation. The purpose of the study was explained to all participants, and written consent was obtained. The study adhered to the core principles outlined in the Declaration of Helsinki.

### Statistical analysis

Data was collected using a structured data entry form after which it was entered into Microsoft Excel software. Statistical analyses were performed on R language for statistical computing^28^. The principal variables analysed included immunohistochemistry (intrinsic subtypes,) and tumour characteristics (such as BCa histological type, tumor size, staging investigations, tumour location, site of lump, skin involvement, and type of skin involvement). Additionally, socio-demographic characteristics (age, marital status, educational background, occupational status, and ethnicity) were considered, along with risk factors (menarche, age at full-term pregnancy, family history of BCa, and the use of combined oral contraceptive pills). Menarche was further categorised into early (12 years and below) and late (more than 12 years). Descriptive statistics were used to examine the distribution of the study variables. Data for continuous variables between two groups were presented as means ± standard deviation (SD). Categorical variables were presented as frequencies (n) and percentages (%). Fisher’s exact test was performed to test the association of various reproductive and clinicopathological factors with pre and postmenopausal groups. A P-value of less than 0.05 was considered statistically significant.

## Results

### Sociodemographic characteristics of the study participants

A total of 336 patients received BCa core biopsy diagnoses between November 2019 and June 2021. Of these, 46 (13.7%) were YPM and 165 (49%) were postmenopausal and 125 (38%) were premenopausal aged 36–49 years. Fifteen YPM and 55 of the postmenopausal women did not meet inclusion criteria. Consequently, 141 eligible YPM women recruited for the study. However, the analysis included data from 140 patients, with one patient (0.07%) excluded due to inadequate data.

Table 1 shows the sociodemographic characteristics of the study participants. Among the participants, thirty-one (22.1%) were aged below 35 years, while 109 participants (77.9%) were identified in the post-menopausal phase. The majority of the participants were married (52.9%), engaged in informal occupations (62.2%) attained a basic education level (37.9%), and belonged to the Akan ethnic group. Similar trends in terms of frequencies of the results were observed between those under 35 years and postmenopausal, except for differences in educational category. While 12 (38.7%) of participants under 35 years had received tertiary education, a majority of postmenopausal participants 44 (40.4%) had completed only basic education level.

**Table 1:** Socio-demographic characteristics of study groups.

Variables	Total	BCa status	
		35 years and below	Post-menopausal
	(n = 140)	(n = 31)	(n = 109)
Marital status			
Divorced	24 (17.1)	0 (0.0)	24 (22.0)
Married	74 (52.9)	20 (64.5)	54 (49.5)
Single	36 (25.7)	11 (35.5)	25 (22.9)
Widowed	6 (4.3)	0 (0.0)	6 (5.5)
Occupational status			
Formal	17 (12.1)	12 (38.7)	5 (4.6)
Informal	87 (62.2)	15 (48.4)	72 (66.1)
Retired	7 (5.0)	0 (0.0)	7 (6.4)
Unemployed	28 (20.0)	3 (9.7)	25 (22.9)
Student	1(0.7)	1 (3.2)	0 (0.0)
Ethnicity			
Akan	121 (86.4)	27 (87.1)	94 (88.7)
Non-Akan	19 (13.6)	4 (12.9)	15 (13.8)
Educational status			
Basic	53 (37.9)	9 (29.0)	44 (40.4)
None	39 (27.8)	3 (9.7)	36 (33.0)
Secondary	27 (19.3)	7 (22.6)	20 (18.4)
Tertiary	21 (15.0)	12 (38.7)	9 (8.3)

BCa: breast cancer.

### Age distribution of below 35 years and postmenopausal participants

**Figure 1:**
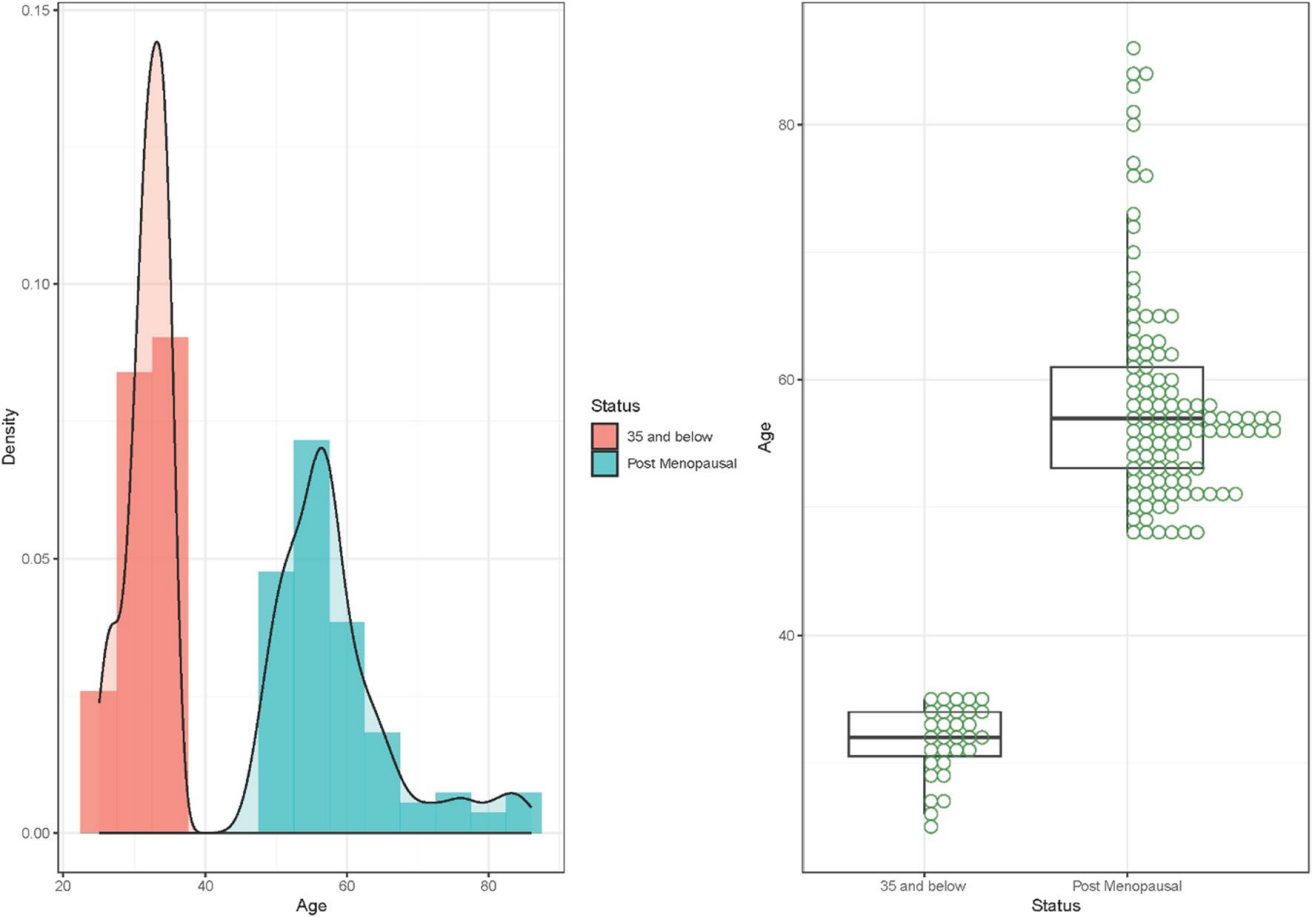
Age distribution of below 35 years and postmenopausal participants.

As shown in Fig. 1, the median age of all participants was 55.0 years (range: 25.0–86.0). The median ages for those below 35 years and postmenopausal were 32.0 (range:25.0–35.0) and 57.0 (48.0–86.0) respectively. A considerable proportion of the study’s participants fell between 31 and 35 years (74.1%) among those under 35 years, while post-menopausal participants were predominantly distributed between 56 and 65 years (48.6%). A few of the participants (3.2%) within the pre-menopausal group were 25 years and below.

### Association of breast-cancer-related risk factors with postmenopausal status

The majority of the under-35 participants (15, 48.4%) and postmenopausal women (46, 42.24%) experienced full-term pregnancy within 19–29 years. The absence of a family history of BCa was prevalent in both groups, with 27 (87.1%) among those under 35 and 95 (87.2%) among postmenopausal women. Similarly, no usage history of oestrogen-containing contraceptive/hormone replacement therapy was found in both groups: 27 (87.1%) and 100 (91.7%). The majority of the participants under 35 years 15 (48.4%) experienced late menarche, while a majority of post-menopausal participants 53 (48.6%) were unsure. Fisher’s exact test revealed no significant correlation between BCa-related risk factors and menopausal status (P > 0.05) (Table 2).

**Table 2:** Association of breast cancer-related risk factors with post-menopausal status.

Variables	BCa status		Fisher’s test
	35 years and below	Postmenopausal	P-value
	n = 31	n = 109	
Menarche			0.150
Early	7 (22.6)	18 (16.5)	
Late	15 (48.4)	38 (34.9)	
Don’t know	9 (29.0)	53 (48.6)	
AFFTP			0.083
Below 19 years	2 (6.5)	14 (12.8)	
19–29 years	15 (48.4)	46 (42.2)	
≥ 30 years	5 (16.1)	7 (6.4)	
Null ip	5 (16.1)	9 (8.3)	
Don’t know	4 (12.9)	33 (30.3)	
Family history of BCa			0.315
Yes	4 (12.9)	8 (7.3)	
No	27 (87.1)	95 (87.2)	
Don’t know	0 (0.0)	6 (5.5)	
Usage of ECHRT			0.484
Yes	4 (12.9)	9 (8.3)	
No	27 (87.1)	100 (91.7)	

BCa: breast cancer; AFFTP: age at first-full term pregnancy; ECHRT: oestrogen-containing contraception/hormone replacement therapy.

### Association of tumour characteristics with postmenopausal women

A greater 107 (98.2%) of the post-menopausal participants had invasive carcinoma. Mean tumour size was significantly higher in those under 35 compared to postmenopausal individuals (8.76 cm ± 6.08 vs. 6.2 cm ± 3.34, P = 0.026). Both groups commonly had Left tumour locations: 51.6% (under 35) and 51.4% (post-menopausal). The upper outer quadrant was the most prevalent tumour site for both groups: 62% of those under 35 years had upper outer quadrant tumor location with 52% in the post-menopausal group. Tumour-related skin involvement was present in 45.2% of patients under 35, all displaying Peau d’orange.) Fisher’s exact test revealed no significant correlation between tumour characteristics and menopausal status (P > 0.05) (Table 3).

**Table 3:** Association of the tumour characteristics with post-menopausal status.

Variables	BCa status		Fisher’s test
	35 years and below below	Postmenopausal	P-value
	n = 31	n = 109	
Tumor size (cm) (mean ± SD)	8.76 ± 6.08	6.2 ± 3.34	0.026
Histological type			0.213
DCIS	2 (6.5)	2 (1.8)	
Invasive carcinoma	29 (93.5)	107 (98.2)	
Staging			0.864
Chest CT and ABD USG	26 (83.9)	92 (84.4)	
Chest Xray and ABD USG	5 (16.1)	15 (13.8)	
Skeletal survey	0 (0.0)	2 (1.8)	
Tumor grade			0.180
Grade one	0 (0.0)	8 (7.34)	
Grade two	15 (48.39)	61 (55.96)	
Grade three	16 (51.61)	40 (36.70)	
Tumour location			0.572
Left	16 (51.6)	56 (51.4)	
Right	15 (48.4)	53 (48.6)	
LVI			0.531
No	1 (3.23)	2 (1.83)	
Yes	30 (96.77)	107 (98.17)	
Site of lump			0.908
Lower inner quadrant	1 (3.2)	5 (4.6)	
Lower outer quadrant	1 (3.2)	10 (9.2)	
More than one quadrant	9 (29.1)	28 (25.6)	
Upper inner quadrant	1 (3.2)	5 (4.6)	
Outer upper quadrant	19 (61.3)	61 (56.0)	
Skin involvement			0.376
No	17 (54.8)	54 (49.5)	
Yes	14 (45.2)	55 (50.5)	
Type of skin involvement			
Peau d’orange	14 (100.0)	37 (67.3)	0.026
Ulceration	0 (0.0)	18 (32.7)	

BCa: breast cancer; Interval, LVI: lymphovascular invasion.

### Distribution and association of clinical stage and cancer subtype with postmenopausal status

**Figure 2:**
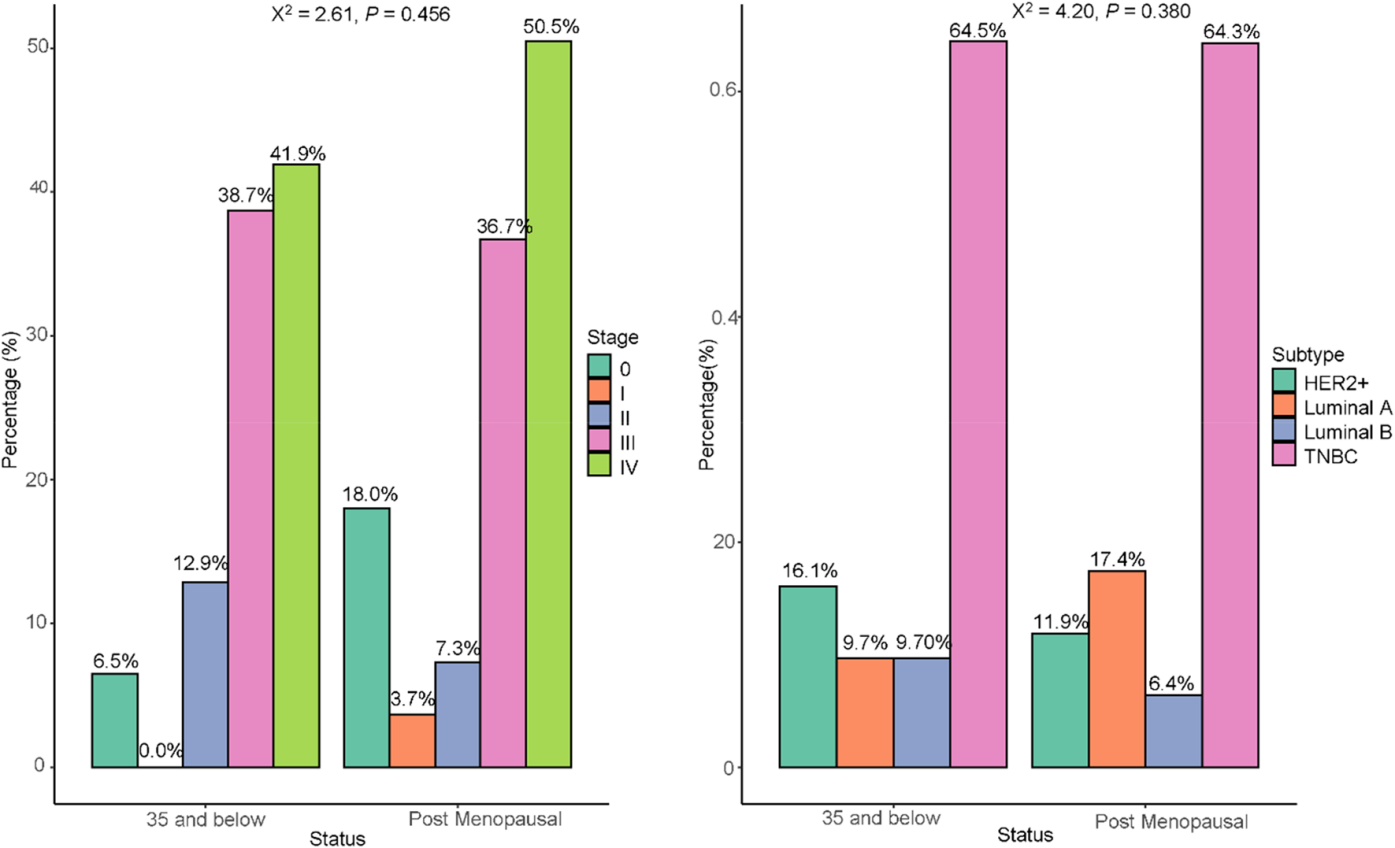
Distribution and association of clinical stage and cancer subtype with postmenopausal.

The majority of the under-35 had stage IV cancer 13 (41.9%) and for postmenopausal individuals, it was 50.4%. None of the participants under 35 had stage I BCa. TNBC subtype was the most expressed subtype 20 (64.52%) followed by HER2+ 5 (16.1%) for under-35 participants. Similarly, for post-menopausal participants, TNBC subtype was the most common subtype 70 (64.3%) followed by luminal A 19 (17.4%). There was no statistically significant association between tumour stages (P = 0.456) and tumour subtypes (P = 0.380) with postmenopausal (Fig. 2).

### Distribution of RECIST response criteria among under 35-year-old and postmenopausal participants

**Figure 3:**
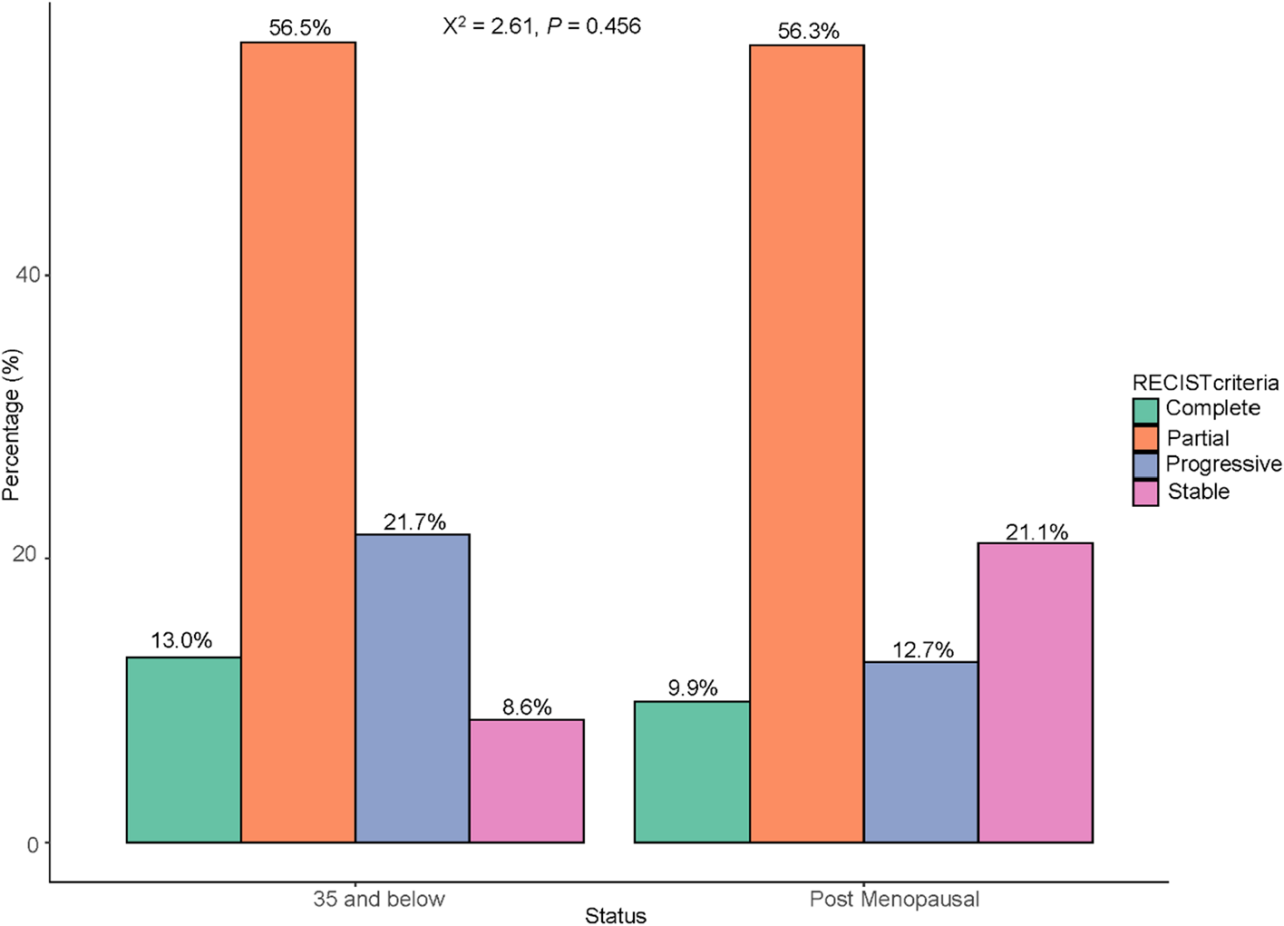
Shows the distribution of RECIST response criteria among under 35-year-old and postmenopausal participants.

Among those under-35, 3 (13.0%) showed complete clinical response, 13 (56.6%) had partial response, 5 (21.7%) had progressive disease and 2 (8.7%) had stable disease. Postmenopausal women exhibited predominantly partial response 40 (56.3%), with 7 (9.9%) achieved complete clinical response and 9 (12.7%) developed disease progression. Stable disease was present in 15 (21.1%) patients (Fig. 3).

## Discussion

BCa accounts for around 16% of all cancer cases in Ghana and is the most common cancer among women^29^. Early-onset BCa may differ in etiologies, clinical features, and outcomes compared with BCa in older women. To gain further insight, we prospectively collected and compared demographic, clinico-pathological features, and outcomes of BCa in women under 35 years and those who have reached menopause at a Ghanaian teaching hospital.

The highest incidence of BCa in Ghana is typically observed between the ages of 40 to 49 years. New cases of BCa in women under 35 years at KATH constitute 13% annually as documented in the Breast Cancer Registry book of 2020. In contrast, the United States had considerably lower figures, with less than 3% for women under 35 and 7% for those under 40^30^. Among 336 women diagnosed with BCa during the study, 13.7% were YPM women, 49.1% were postmenopausal, and 37.2% were premenopausal. Similar patterns were observed in a study in Ghana by Clegg-Lamptey et al., showing 8% for YPM, 52% between ages 35–48 years, and 40% above 50 years^21^. Moreover, other regions have reported a higher proportion of BCa cases among women under 35 years, Togo, and Senegal, for instance, have reported 38.3% and 22.6%, respectively^16,17^. Ntekim et al. found 30.6% of BCa cases in women aged 15 to 39 years in Nigeria^31^ while Ntirenganya et al. also reported a proportional rate of 45.9% of BCa cases in women below 40 years in Rwanda^32^. The incidence of BCa in YPM women is higher in Africa as compared to other parts of the world. Hwang et al. found 4.4% of women under 35 years in South Korea^33^, Eiriz et al. recorded 2.5% of women under 35 in Portugal^11^, and Jmor et al. reported 2.0% for women under 35 in Edinburg^34^, The disparities may in part be explained by the population age structures and the prevalence of younger populations in African countries.

Among young premenopausal in this study, a significant portion were married, and none were divorced or widowed. Among postmenopausal, 49.5% were married, approximately half were either single or divorced, with a small fraction widowed (5%). Similar marital status distributions were reported in an Accra-based study by Iddrisu et al. for pre- and postmenopausal women with BCa in Ghana^35^. A study by Hinyard et al. marital status and BCa-related outcomes suggested that younger unmarried BCa women may benefit from additional counseling at the time of diagnosis^36^. While marriage reduces BCa and metastasis risk, it can present psychosocial challenges for young married couples^37,38^.

Reproductive history has been consistently associated with BCa risk^39,40^. Age at menarche, age at first full-term pregnancy, and nulliparity are well-established risk factors^40–42^. However, their specific impact on pre- and postmenopausal BCA risk varies across studies^43^. This study found that the majority of under-35 and postmenopausal women had full-term pregnancies between 19 and 29 years, and many under-35 participants had late menarche. The findings indicated there were no differences in the distribution of reproductive risk factors for BCa in both groups in this study. Both groups also had a high prevalence of no family history of BCa, consistent with reports from the literature, as most BCa patients lack a family history^44^. Experts caution against overemphasizing hereditary factors to avoid false security in some women^45^.

In this study, the commonest pathological type of BCa among YPM and postmenopausal women was invasive carcinoma at 91.67% and 98.17% respectively. These results are consistent with earlier Ghanaian studies which documented comparable findings ranging from 82 to 86%^20–22^. Moreover, these findings also mirror reports from a retrospective study by Eiriz et al.^11^ where in situ disease accounted for 6.45% of YPM women and 1.03% of postmenopausal women. The average tumor size at diagnosis indicated an advanced-stage disease measuring 8.76 cm (T3) for YPM women and 6.20 cm (T3) for postmenopausal women. Clegg-Lamptey et al. found an average tumour size of 6 × 7 cm in southern Ghana^21^ while another study in Tanzania reported an average tumour size of 6 cm^46^.

Most breast cancer lumps for both age groups were located in the upper outer quadrant followed by involvement in multiple quadrants. This result is consistent with reports from previous studies in Ghana^20–22^. The increased prevalence in the upper outer quadrant may be attributed to the amount of target epithelial tissue in that region, but other studies have suggested that causative factors may play a role^47^. The study revealed a minimal predilection for the left breast with a left breast/right breast ratio of 1.07 in both groups similar to results from the USA, Europe, and other African countries. Attributable reasons so far are inconclusive^48^.

Both YPM and the postmenopausal group showed concerning rates of advanced stage BCa with 80.65% and 87.1% presented at stages III and IV, respectively. The clinical stage at presentation did not differ between the two groups. Similar trends have been observed in previous studies in Ghana^20–22^ and many other sub-Saharan African countries^49,50^, indicating a persistent problem of late-stage diagnosis. Efforts to improve breast cancer awareness and education have not led to better early diagnosis rates. Asoogo et al. conducted a study in BCa presenting at KATH and identified key factors contributing to the late presentation including insufficient disease knowledge fear of mastectomy and cancer treatment, poverty, and destructive cultural beliefs^51^.

TNBC is more prominent in premenopausal African American and African women compared to women of European descent^52,53^. In this study, 64.5% of the YPM had TNBC while 16.3% had the HER2 + subtype. Postmenopausal women showed similar proportions 64.2% for TNBC and 11.9% for HER2 subtype and no statistical difference between the two groups. These findings concord with results from a retrospective study done by Mensal et al. in Ghana which high TNBC prevalence (66.38%) and 9.5% for the HER2+ subtype^54^. A comparative study by Jiagge et al. found that in patients under 50 years, TNBC was most prevalent in (50.8%) and African Americans (34.3%) compared with White Americans (16.0%) and Ethiopians (16.0%)^55^. Follow-up population-based studies supported the link between WA ancestry, increased TNBC rates, and more unfavourable disease outcomes^55^. Additionally, different studies from Nigeria (25.0%) and Kenya (20.3%) have also reported a high prevalence of TNBC^56,57^. Biologic and environmental factors may contribute to racial disparities in TNBC^52^. Tumour grade among young premenopausal women was either 48.39% for grade 2 or 51.61% for grade 3. 96.77% had no lymphovascular invasion. Among postmenopausal patients, tumour grade 2 was 55.96% and grade 3 was 36.70% and 98.17 had no lymphovascular invasion. Similar findings have been reported in different studies in sub-Saharan Africa^56,58^.

In this study, 13.0% of YPM women achieved complete clinical response, with 56.6% showing a partial response, but 21.7% progressed, and 8.7% remained stable. These findings are comparable to reports study by Egwuonwu et al.^59^, where the premenopausal response revealed 12.9% complete clinical response, 61.3% partial response, and 25.8% non-response. In postmenopausal women, 56.3% showed partial response, and 9.9% demonstrated a complete clinical response. However, 12.7% experienced disease progression and 21.1% showed stable disease. These findings highlight the aggressive nature and high progression of BCa in the younger population. Furthermore, among YPM who had either complete or partial response to anthracycline-based neoadjuvant chemotherapy, 80.95% had TNBC or HER2 subtypes, while 19.1% had luminal breast cancer For postmenopausal women with either complete clinical response or partial response, 77.42% had TNBC or HER2+ subtype and 22.6% Luminal A and B These results are consistent with reports from a study done by Carey et al. indicating that Basal-like and HER- molecular subtypes are more sensitive to anthracycline-based neoadjuvant chemotherapy than luminal breast cancers^60^.

The study had some limitations, including its single-centre design and small sample size. The reported numbers likely represent gross underestimate of the true prevalence of BCa among YPM and postmenopausal women in Ghana, given that a majority of patients never make it to the hospital for diagnosis. Moreover, the limited sample size undermines the suitability of robust statistical testing. The study did not evaluate loss of androgen-reception expression which has been shown to be associated with aggressive BCa in women of African descent, nor did it examine immune markers of exhaustion. Nevertheless, our findings are congruent with previous reports and highlight some significant demographic and clinical characteristics of BCa in YPM and postmenopausal women.

## Conclusion

The study highlights consistent tumour characteristics in both YPM and postmenopausal women with a higher prevalence of TNBC in both groups. Advanced clinical stages at diagnosis were common. Anthracycline-based neoadjuvant chemotherapy was effective for TNBC and HER2+ subtypes. YPM women often experience more disease progression. Establishing Breast Care Clinics in district and regional hospitals for early detection is crucial. Further studies are warranted to understand the higher prevalence of TNBC in black Africans and re-evaluate breast education programs to address persistently late presentations.

## Data Availability

The data used to support the findings of this study will be made available upon request to the corresponding author.
